# Green Synthesis and Characterization of Xanthium strumarium-Mediated Titanium Dioxide Nanoparticles

**DOI:** 10.7759/cureus.51012

**Published:** 2023-12-23

**Authors:** Shravani V P, Shantha K Sundari, Sivakamavalli Jeyachandran, Shweta Nagesh

**Affiliations:** 1 Orthodontics and Dentofacial Orthopaedics, Saveetha Dental College and Hospitals, Saveetha Institute of Medical and Technical Sciences, Saveetha University, Chennai, IND; 2 Laboratory in Biotechnology and Biosignal Transduction, Saveetha Dental College and Hospitals, Saveetha Institute of Medical and Technical Sciences, Saveetha University, Chennai, IND

**Keywords:** research, species, xanthium strumarium, cytotoxicity, nanoparticles, plants

## Abstract

Background

Green synthesis of nanoparticles is a growing trend. The annual plant *Xanthium strumarium* L. (*X. strumarium*) belongs to the Asteraceae family. The herb has traditionally been used to treat a variety of ailments, including leucoderma, dangerous insect bites, epilepsy, salivation, allergic rhinitis, sinusitis, etc. Inorganic, biocompatible, and non-toxic titanium is a substance employed in the pharmaceutical and biomedical industries as well as in fields like bone tissue engineering. The aim of the study is to characterize titanium dioxide nanoparticles (TiO₂NPs), which were synthesized from *X.strumarium*. Also, this study aims to assess the cytotoxic properties of the synthesized leaf extract and the TiO₂NPs.

Materials and methods

In this study, the biosynthesis of TiO₂NPs was made from *X. strumarium* leaf extract. The characterization of the green-synthesized TiO₂NPs was done using the spectral analysis of an ultraviolet (UV)-visible spectrophotometer, scanning electron microscopy (SEM), and Fourier Transform Infrared Spectroscopy (FTIR). The advantage of using TiO₂NPs is that they possess antimicrobial, antibacterial, chemical stability, and catalytic properties. The leaf extract and the biosynthesized nanoparticles were tested against human fibroblast cell lines for biocompatibility using 3-(4,5-dimethylthiazol-2-yl)-2,5-diphenyl-2H-tetrazolium bromide (MTT) assay.

Results

SEM investigation showed that TiO₂NPs were crystalline in nature. FTIR confirms the presence of alkyne and amine functional groups, and the pointed vertices in the X-ray diffraction (XRD) pattern show the crystalline nature of TiO2NPs. The study found that the cell viability of TiO₂NPs was 110%.

Conclusion

TiO₂NPs were synthesized from *X. strumarium* leaf extract and characterized using SEM, FTIR, and XRD. The TiO₂NPs were found to be crystalline in nature with various functional groups. MTT assay shows that the synthesized nanoparticles are promising biocompatible agents that can be used in future research in the medical field.

## Introduction

The field of nanotechnology includes the formation, characterization, and use of materials with nanoscale dimensions. Specific features of nanoparticles can be attributable to their extremely small size of 1 to 100 nm, which can provide a high surface area-to-volume ratio [1].

*Xanthium strumarium* L. (*X.strumarium*), also known as cocklebur or burweed, is typically seen as a weed in rice fields and hedges across the tropical regions of India [2]. These leaf extracts have been shown to have antifungal, anti-inflammatory [3], anti-leishmanial [4], anti-trypanosomal [5], hypoglycemic [6], anti-ulcerogenic [7], diuretic [2], and anticancer [8] effects. When compared to the drug's traditional forms, drugs bound to nanoparticles are anticipated to have a number of beneficial effects [9].

Most metal and metal oxide nanoparticles were previously created using various physical and chemical techniques [10]. Because of the environmentally friendly products, biocompatibility, and long-term economic sustainability of dependable biosynthetic approaches, especially in the medical industry, they have gained great relevance. Titanium dioxide (TiO₂) nanoparticles (NPs) are highly photostable and non-toxic [11,12]. TiO₂ has uses in the pharmaceutical and biomedical sciences, including bone tissue engineering. The antimicrobial, antibacterial, chemical stability, and catalytic properties of TiO₂NPs make them suitable for use in industrial products such as pigments, fillers, catalyst supports, and photocatalysts [13-17]. Using titanium dioxide (TiO₂) in the production of biological implants has been made possible by its lightweight nature and resilience to corrosion. TiO₂NPs' surface morphology with nano-topography is crucial for osseointegration, which favorably affects osteoblast adhesion, proliferation, and differentiation [18]. Although literature exists on the synthesis of TiO₂NPs and the use of plant extracts for NP synthesis, the combination of *X. strumarium *as a mediator and the chosen characterization techniques distinguishes our study. The aim of the present study is to contribute valuable insights into the characterization and potential cytotoxic effects of this unique combination, thereby advancing the current understanding in the field. The primary objective of our study, which is to characterize and assess the cytotoxic properties of both the synthesized leaf extract and the TiO₂NPs, is not only distinctive but also aligns with the growing interest in understanding the biological effects of nanoparticles.

## Materials and methods

Extract preparation 

*X.strumarium* leaves were procured from a plant nursery in Chennai (Lattitude: 13.0827° N, Longitude: 80.2707° E), India. The fresh leaves were collected and washed thoroughly with cold water and distilled water, the leaves were allowed to dry for five to seven days at room temperature, and fine powder was prepared by using a mechanical grinder. Two grams of the fine powder were mixed with 200 ml of de-ionized water and boiled for 30 minutes at 100°C using a water bath. To remove the solvent from the extract, the filtrate was concentrated at 50°C for 30 minutes after being filtered with Whatman Grade No. 1 filter paper (Cytiva, Marlborough, Massachusetts, United States). The extract was stored in a refrigerator (at 4°C) for further analysis and the preparation of NPs [19].

NPs synthesis

The 0.1 M TiO₂ solution was prepared by adding distilled water and 1.69 g of titanium oxide in volumetric flasks. The titanium oxide and water were continuously stirred and more distilled water was added. The volume was made up to 100 mL, and mixed thoroughly. The solution was kept for at least one hour and then used for further processes. In this study, the leaf extract of *X. strumarium* was used to create TiO₂NPs utilizing a green synthesis method [20].

Ultraviolet-visible (UV-vis) spectra analysis

The UV-vis spectral analysis of synthesized (chitosan-silver) Cs-Ag NPs was done using the Shimadzu UV-1800 spectrophotometer (Shimadzu Corporation, Kyoto, Japan). The spectrophotometer operates as a diagnostic test that serves the purpose of identifying variations among samples by isolating and exploiting their color signatures. The presence of *X. strumarium *TiO₂NPs was confirmed by measuring the wavelength in the range of 200-800 nm [21,22].

Fourier transform infrared (FT-IR) analysis 

FT-IR evaluation was done to determine the possible functional group liable for the reduction of Ti ions, and the infrared spectra were recorded in the wavelength interval of 4000 to 400 cm^-1^ (ALPHA II Compact FT-IR Spectrometer, Bruker Corporation, Billerica, Massachusetts, United States) [23].

X-ray diffraction (XRD) analysis 

The synthesized silver NPs were studied with XRD. The XRD lattice was documented using a computational XRD system, model JPX-8030 (JEOL, Ltd., Tokyo, Japan), with Copper K-alpha (CuK-α) radiation in the range of 20 Å at 40 kV. The XRD peak was analyzed using Diffrac Suite EVA software (Bruker Corporation, Billerica, Massachusetts, United States). The size of the TiO₂NPs was measured from the XRD peak positions using Bragg’s law [24].

Scanning electron microscopy (SEM) analysis

A SEM investigation was performed using a SEM apparatus (JEOL, Ltd., Tokyo, Japan). Thin films of the sample were dropped on a carbon grid, the additional solution was cleared using blotting paper, and the films on the SEM grid were dried under a mercury lamp for five minutes [25].

Human gingival fibroblast (HGF) cells

Eagle minimum essential medium F12 containing 15% (vol/vol) heat-inactivated fetal bovine serum (FBS), 2 mM L-glutamine, 50 IU/mL penicillin, and 50 mg/mL streptomycin was used to cultivate human gingival fibroblast (HGF) cells under standard incubation conditions (37°C, 95% air/5% carbon dioxide (CO2)) until they reached confluence (70-80%). The cells were separated using trypsin solution after one week and then replated in 6-well plates with a cell density of 5.0 x 105 per well. Following a 24-hour period of cell attachment, two milliliters of full Dulbecco's Modified Eagle Medium (DMEM) F-12 medium were added to each well.

MTT (3-(4,5-dimethylthiazol-2-yl)-2,5 diphenyl tetrazolium bromide) assay

One milliliter of complete culture media was placed in each well of the 6-well plate. The bottom well then received 0.5 mg/mL MTT. Following incubation at 37°C for four hours, the culture medium was aspirated from the well. The formed formazan crystals were solubilized by adding 100µl of dimethyl sulfoxide (DMSO) solution to each well. The cell types were shaken to evenly combine the blue reaction product with the solvent. Then, 100µl of the colored DMSO was transferred from each well to a 96-well plate for quantifying the cell viability. Absorbance at 450 nm was measured using a microplate reader.

## Results

The absorption spectra of the synthesized *X. strumarium* TiO₂NPs were analyzed in the UV-vis spectrophotometer. The color of the solution was changed from yellow-brown to dark brown. The color-changed solution was examined with the UV-vis spectrophotometer. The analysis revealed a series of peaks between 300 nm and 600 nm. The presence of *X. strumarium* TiO₂NPs was detected by surface plasmon resonance (SPR).

**Figure 1:**
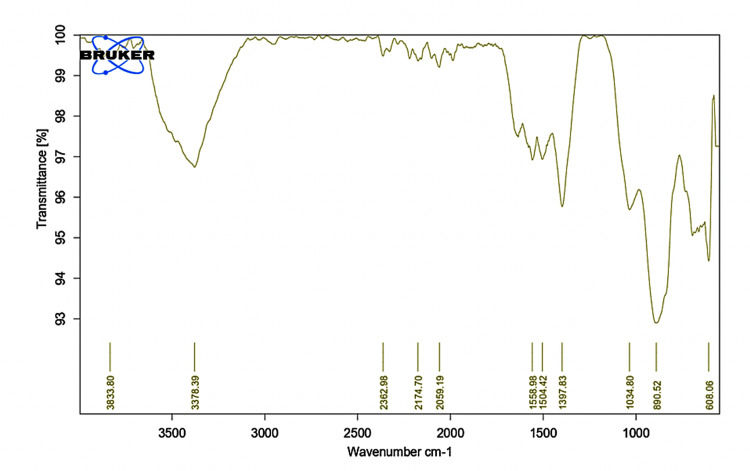
FT-IR spectra of as-synthesized X. strumarium TiO₂NPs FT-IR: Fourier transform infrared; X. strumarium: Xanthium strumarium L.; TiO₂NPs: titanium dioxide nanoparticles

The analysis of distinct functional groups within TiO₂NPs was conducted using FT-IR spectroscopy. Figure 1 illustrates the FT-IR spectra of *X. strumarium*-mediated green synthesized TiO₂ within the 400-4000 cm⁻¹ range. The bands were observed at 3833, 3378, 2362, 2174, 2059, 1558, 1504, 1397, and 1034. The spectral peaks at 3833.80 and 3378.39 cm⁻¹ correlate to the stretching vibrations of the OH (hydroxy) group. In the pure TiO₂ spectrum, distinctive peaks at 608.06 cm⁻¹ signify the stretching vibration of Ti-O bonds, while peaks at 1397.83 cm⁻¹ denote stretching vibrations of Ti-O-Ti bonds. Moreover, the presence of amines is indicated by peaks at 3378.39 cm⁻¹, alkynes at 2362 cm⁻¹, aromatic rings at 2059 cm⁻¹, pyridines at 1558.98 cm⁻¹, and thiophenes at 1054.80 cm⁻¹ in the FT-IR spectrum.

**Figure 2:**
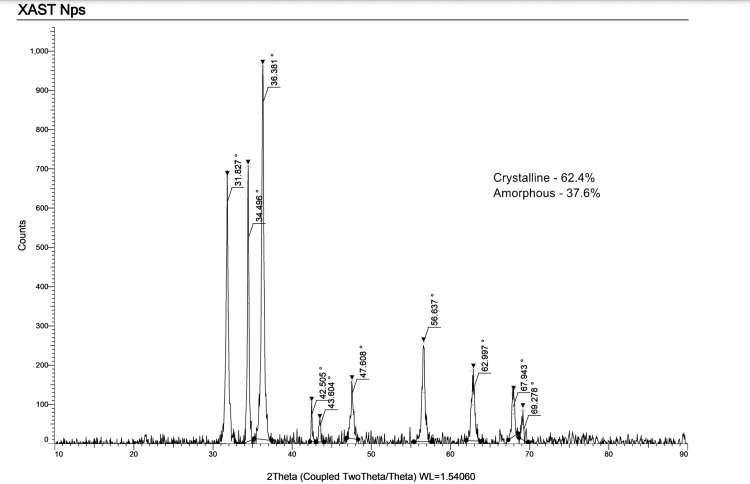
XRD spectra of the synthesized X. strumarium-mediated TiO2 NPs. XRD: X-ray diffraction; X. strumarium: Xanthium strumarium L.; TiO₂NPs: titanium dioxide nanoparticles

X-ray diffraction measurements supported the presence of TiO₂NPs synthesized using *X. strumarium* leaf extract. XRD evaluation revealed seven definite diffraction peaks at 31.3°, 34.8°, 36.9°, 42.5°, 43.8°, 47.5°, and 56.1°, which recorded the plane at 400, 390, 580, 100, 90, and 280 of the cubic face-centered TiO₂, respectively (Joint Committee on Powder Diffraction Standards (JCPDS) No. 21-1272). Using Scherrer's formula, the mean grain size formed during biosynthesis was estimated to be 100 nm for the more intense peak, d = 0.89/cos. The presence of sharp peaks confirmed the crystalline nature of the synthesized nanoparticles (Figure 2). 

**Figure 3:**
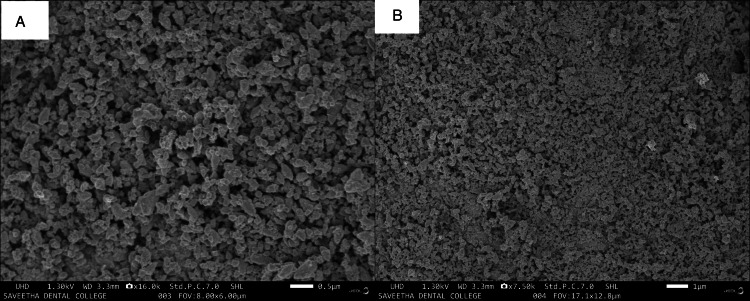
A) Scanning electron microscope image of as-synthesized X. strumarium TiO2NPs, 0.5 um magnification; B) scanning electron microscope image of as-synthesized X. strumarium TiO2NPs, 100 um magnification. X. strumarium: Xanthium strumarium L.; TiO₂NPs: titanium dioxide nanoparticles

The surface morphology of TiO₂NPs was examined using scanning electron micrographs (SEM, JEOL, Ltd., Tokyo, Japan). An SEM micrograph of TiO₂NPs with a diameter of 200 nm is shown in Figure 3; specific TiO₂NPs had several structures such as pentagons, irregular spheres, and hexagons.

## Discussion

The present study characterized *X. strumarium-mediated* titanium dioxide nanoparticles using SEM, FTIR, and XRD. The findings show that the synthesized NPs were crystalline in nature containing various functional groups. Also, XRD results show the purity of the NPs synthesized. In a study by Shanavas et al. [26], the formation of spherical-shaped TiO₂NPs was evident in SEM and TEM analysis. In our study, FT-IR evaluation revealed the presence of alkynes, aromatic rings, and amine functional groups. The study done by Rajakumar et al. [27] synthesized TiO₂Nps using *Eclipta prostrata* leaf aqueous extract and reported FT-IR spectra band peaks at 3410-1, 1621-1, 1368-1, 1077-1, and 1 065 cm-1, unlike the present study. This can be attributed to the possible involvement of alcohols (OH), asymmetrical stretch, primary amines, aromatics, and aliphatic amines in the synthesis of TiO2NPs using *X. strumarium* leaf extract. Functional groups corresponding to these are responsible for the bioreduction of TiO(OH)_2_ to TiO₂NPs.

In our study, the cell viability of HGF cells treated with TiO₂NPs was 110% whereas for the HGF cells treated with the leaf extract of *X. strumarium*, the cell viability was 80%. This shows the cytotoxic potential of both the extract and the nanoparticles synthesized. In a study by Eslami et al., at minimum inhibitory concentration (MIC), the mean cell viability in the TiO₂ group was considerably higher compared to zinc oxide, copper oxide, silver nanoparticles, and chlorhexidine. The cell viability of TiO_2_NPs was similar to the values in the present study [28]. In another study, the TiO_2_Nps in all concentrations were not found to be cytotoxic [29]. TiO_2_NPs were tested for cytotoxicity in mouse macrophage Ana-1 and MH-S cells in a study by Zhang et al. [30], and it was concluded that the TiO_2_NPs showed fewer toxic effects, especially in MH-S cells, and it was shown that the toxic impact was dependent on the size and structure of the particles. According to Wang et al. [31], ultrafine TiO_2_ can cause genotoxicity and cytotoxicity in cultured human cells. Furthermore, Hussain et al. [32] reported TiO_2_NPs-induced cytotoxicity in rat liver cells. Park et al. [33], showed dose-related apoptotic damage on human lung epithelial (A549) cells after being treated with TiO2 nanoparticles. The present study found that TiO_2_NPs synthesized using *X. strumarium* showed improved cytotoxic activity.

Limitations

The current investigation evaluated the cytotoxic properties of TiO_2_NPs produced solely from *X. strumarium* leaf extract. Different preparation methods and the cytotoxic effects might be compared to support the optimal synthesis process. Further studies using the leaf extract and the synthesized nanoparticles against different cell lines must be done. The shelf life of the green-synthesized *X. strumarium* leaf extract is influenced by several factors, including storage conditions and the absence of adverse environmental elements. Further investigation is needed regarding the various properties of *X. strumarium-*mediated TiO_2_NPs, for its utility as a biomedical agent.

## Conclusions

The current study characterizes the structural and morphological properties of the TiO2NPs synthesized using the leaf extract of *X. strumarium*. The nanoparticles were shown to be crystalline in nature with different functional groups. The present study found that TiO2NPs synthesized using *X. strumarium* showed improved cytotoxic activity. Hence, it was revealed that both the leaf extract and the synthesized NPs are promising biocompatible agents and that these can be used for further studies in the medical field.
